# Fast semi-automated analysis of pulse wave velocity in the thoracic aorta using high temporal resolution 4D flow MRI

**DOI:** 10.1186/1532-429X-15-S1-P87

**Published:** 2013-01-30

**Authors:** Bruce Spottiswoode, Aurelien F Stalder, Mehmet A Gulsun, Karissa F Campione, Maria Carr, Marie Wasielewski, Michael Markl

**Affiliations:** 1 Cardiovascular MR R&D, Siemens Healthcare, Chicago, IL, USA; 2 Magnetic Resonance, Imaging & Therapy, Healthcare Sector, Siemens AG, Erlangen, Germany; 3 Corporate Technology, Siemens Corporation, Princeton, NJ, USA; 4 Radiology, Northwestern University Feinberg School of Medicine, Chicago, IL, USA; 5 Biomedical Engineering, Northwestern University, Chicago, IL, USA

## Background

Pulse wave velocity (PWV) gives an indication of vessel stiffness, which can be used as a marker of age related changes in compliance, as well as to assess changes in vessel elasticity as a measure of atherosclerosis [1,2]. A recent meta-analysis showed PWV to be a robust predictor for cardiovascular events and all-cause mortality [3]. Recently, 4D flow MRI has been applied to assess PWV with full volumetric coverage of the aorta, but the analysis was limited by its low temporal resolution and the labor intensive segmentation of multiple analysis planes along the aorta. In this study, we propose a fast and semi-automated method for reliably extracting PWV from 4D flow data.

## Methods

Six normal volunteers were scanned on a 3T MRI system (MAGNETOM Skyra, Siemens AG, Erlangen) with informed consent and IRB approval. Navigator gated 4D flow data [4] of the thoracic aorta were acquired with three directional velocity encoding, venc = 150 cm/s, voxel size = 2.3 x 2.3 x 2.3 mm^3, a k-t GRAPPA [5] acceleration factor of R = 5, temporal resolution = 20 ms, and a scan time of 10-15 minutes.

The data were imported into an investigational 4D Flow Evaluation Tool [6] (Siemens AG, Erlangen). Background phase correction and vessel tracking were applied, followed by a semi-automated center line extraction and aortic lumen segmentation [7,8]. One hundred evaluation planes were then automatically reconstructed along the center line, and flow-time curves were automatically calculated for each plane based on the segmentation contours. The center line coordinates and flow waveforms were imported into a custom MATLAB (Mathworks, Natick, MA) tool, where PWV was derived from the data automatically by fitting a plane to the upslope of all flow waveforms [9].

## Results

**Figure 1:**
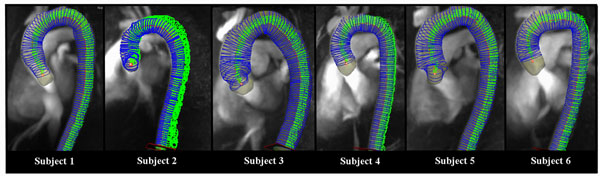
Center line and evaluation planes derived using the investigational 4D Flow Evaluation Tool (Siemens AG, Erlangen).

**Figure 2:**
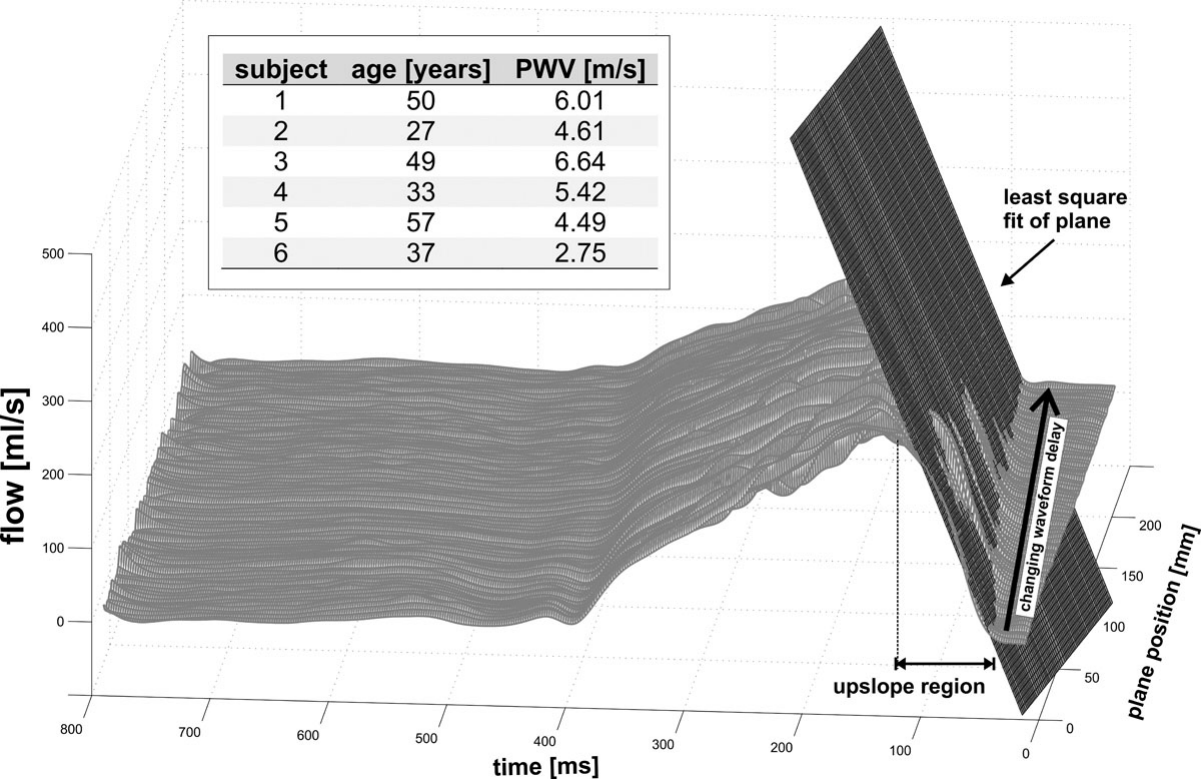
Pulse wave velocity estimation based on flow-sensitive 4D MRI data. Global PWV was estimated by fitting a plane to the upslope region of the data from all slices to quantify the changing waveform delay from the ascending to the descending aorta. The table summarizes the estimated PWV's for all subjects included in the volunteer study.

Figure 1 shows that 100 evenly spaced analysis planes could be successfully prescribed from the vessel center line for all 6 subjects. The analysis could be performed in a short time (5 - 10 minutes) and included only minimal user interaction (manual identification of proximal ascending and distal descending aorta). As illustrated in Figure 2, fully automated analysis based on the detection of the early systolic upslope region in all waveforms enabled the estimation of global aortic PWV's which correspond well with the literature [1-3,9].

## Conclusions

Using highly accelerated k-t GRAPPA, 4D flow data covering the heart and great vessels can be acquired with improved temporal resolution in a reasonable scan time. Using vessel centerline detection and early systolic upslope detection algorithms, it is possible to reliably extract PWV from 4D flow data in a rapid and semi-automated manner.

## Funding

Grant support by NIH R01HL115828; Dixon Translational Research Grant Initiative, Northwestern Memorial Foundation.
